# Ultrasound-guided interlaminar approach for nusinersen administration in patients with spinal muscular atrophy with spinal fusion or severe scoliosis

**DOI:** 10.1186/s13023-023-02630-8

**Published:** 2023-02-17

**Authors:** Cuijie Wei, Zhenwei Liang, Ying Wu, Shan Liu, Jianxing Qiu, Lingchao Meng, Chunde Li, Shuang Li, Xinhua Bao, Zhaoxia Wang, Luzeng Chen, Hui Xiong

**Affiliations:** 1 Department of Pediatrics, Peking University First Hospital, No. 1, Xi’anmen Street, West District, Beijing, 100034 China; 2 Department of Ultrasonography, Peking University First Hospital, No. 7, Xishiku Street, West District, Beijing, 100034 China; 3 Department of Neurology, Peking University First Hospital, Beijing, 100034 China; 4 Department of Orthopedic/Spine Surgery, Peking University First Hospital, Beijing, 100034 China; 5 Department of Radiology, Peking University First Hospital, Beijing, 100034 China; 6 Beijing Key Laboratory of Molecular Diagnosis and Study on Pediatric Genetic Diseases, Beijing, 100034 China

**Keywords:** Ultrasound guidance, Spinal muscular atrophy, Spinal fusion, Scoliosis, Nusinersen

## Abstract

**Background:**

Intrathecal injection of medications can be challenging in spinal muscular atrophy (SMA) patients with severe scoliosis or after spine surgery. Here we report our experience with real-time ultrasound (US)-guided intrathecal administration of nusinersen in patients with SMA.

**Methods:**

Seven patients (six children and one adult) with either spinal fusion or severe scoliosis were enrolled. We performed intrathecal injections of nusinersen under US guidance. The efficacy and safety of US-guided injection were explored.

**Results:**

Five patients had undergone spinal fusion, while the other two presented severe scoliosis. Success was achieved in 19/20 lumbar punctures (95%), 15 of which were performed through the near-spinous process approach. The intervertebral space with a designated channel was selected for the five postoperative patients, while the interspaces with the smallest rotation angle were chosen for the other two patients with severe scoliosis. In 89.5% (17/19) of the punctures, the number of insertions was no more than two. No major adverse events were observed.

**Conclusion:**

Given its safety and efficacy, real-time US guidance is recommended for SMA patients with spine surgery or severe scoliosis, and the near-spinous process view can be used as a interlaminar puncture approach for US guidance.

**Supplementary Information:**

The online version contains supplementary material available at 10.1186/s13023-023-02630-8.

## Background

Nusinersen was the first approved disease-modifying therapy for spinal muscular atrophy (SMA), and it effectively improves motor function [1]. Given its large molecular weight, it cannot pass the blood–brain barrier, thereby requiring an intrathecal injection. However, the intrathecal injection can be challenging for patients with SMA with severe spinal deformities or spondylodesis. Computed tomography (CT) guidance, cone-beam CT guidance or fluoroscopy guidance can facilitate lumbar punctures [2–5], but they can cause accumulated radiation exposure during long-term applications [6], especially for children, leading to potential cancer risks [7]. Ultrasound (US) guidance has the advantages of nonradiation and real-time administration [8], but owing to technical challenges, it is seldom used for intrathecal injection in patients with SMA with scoliosis [9–13] and even more seldom in patients after spine surgery [11, 12].

In this study, we report our experience with real-time US-guided intrathecal injections of nusinersen in patients with SMA after spinal fusion or patients with severe spinal deformities. We also provide detailed technical points to encourage the use of US during intrathecal nusinersen administration.

## Methods

### Study population

The Ethics Committee of Peking University First Hospital approved this study. From January 1, 2022 to June 30, 2022, 50 patients genetically diagnosed with SMA were admitted to our hospital for treatment with nusinersen. However, seven of them had severe scoliosis and/or spinal surgery, which resulted in difficulty with lumbar puncture. Before the first attempt, the possible risks and benefits of US guidance and CT guidance were discussed with each patient and their guardians. All of them chose US-guided lumbar puncture and signed an informed consent form.

### Preparation before intrathecal injections

We collected patients’ clinical data such as age during symptom onset and initial treatment, sex, SMA subtype, motor function, and genotype. For postoperative patients, we reviewed the detailed procedures of spine surgery, including the type and extent of surgery and the location of the channel reserved for lumbar puncture. Before the first intrathecal injection, anteroposterior and lateral full-body X-rays were taken to analyze Cobb’s angle and the degree of lumbar spine rotation, which was assessed according to Nash and Moe criteria [14]. Unlike previous reports [9, 11, 13], we did not routinely perform spinal three-dimensional computed tomography (3D-CT) in this study to reduce radiation exposure.

### US-guided intrathecal delivery of nusinersen

Additional file 1 (Video) details the entire intrathecal injection procedure. The lumbar puncture operators were sonographers with experience in US-guided puncture (one senior physician with 20 years of experience and two young physicians with 7 and 5 years of experience). All patients were placed in the left lateral decubitus position, with the hip and knee flexed as much as possible to widen the lumbar intervertebral space.

**Fig. 1:**
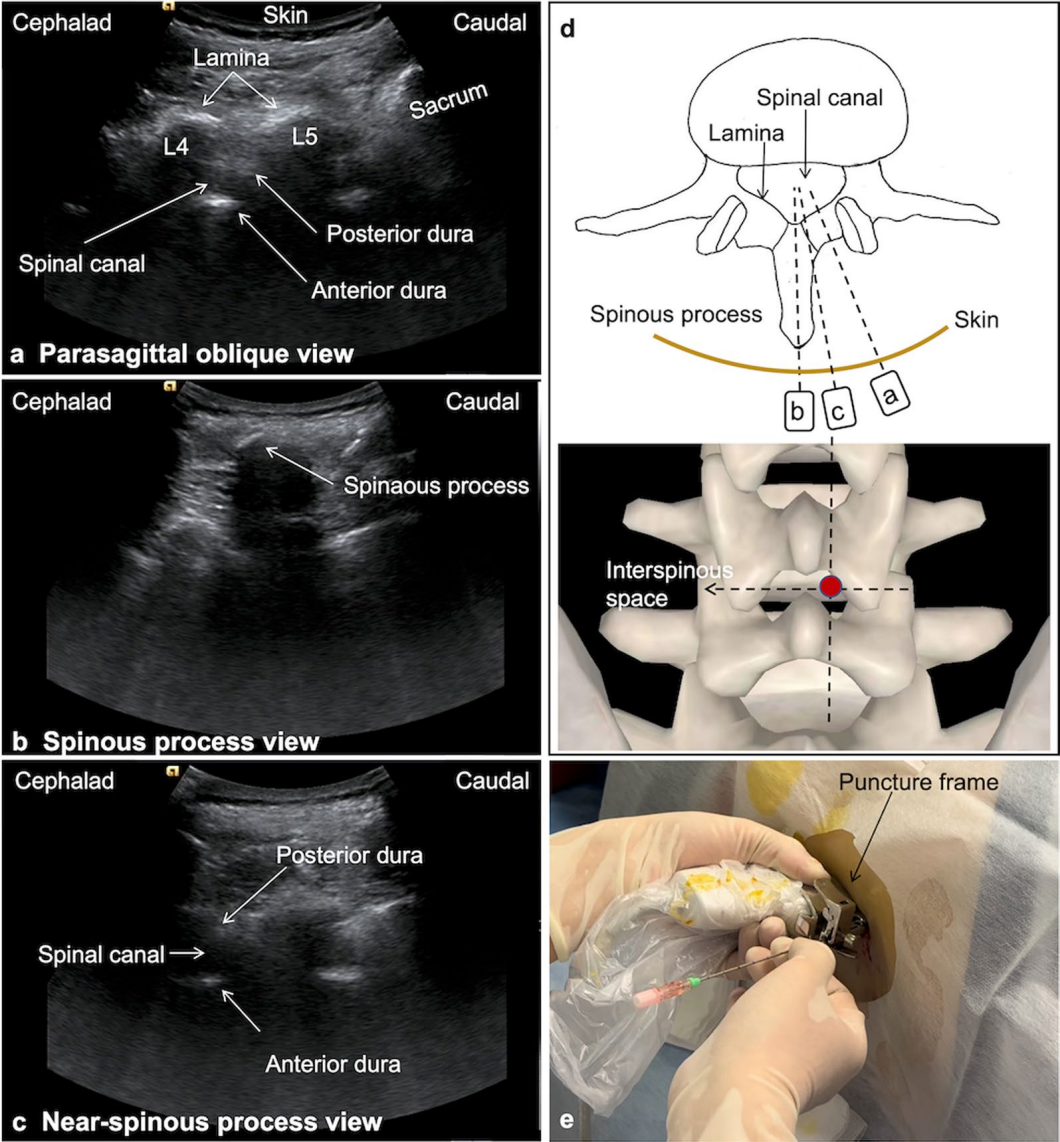
Different sagittal views under ultrasound. **a** Parasagittal oblique view. **b** Spinous process view. **c** In the near spinous process view, the spinal canal is clearly shown without bony structures. **d** Schematic diagram of the vertebral body shows the position of the different views in the transverse section, as well as the location of the puncture point (red dot). **e** During the puncture process, the left hand held the probe for real-time guidance, and the right hand inserted the needle along the fixed angle on the guide frame

US scanning was performed using a Canon Aplio 800 system (Canon Medical Systems, Tokyo, Japan) with an i8CX1 convex array probe (1–8 MHz). Before lumbar puncture, the interlaminar space of interest and optimal puncture route were determined through preprocedural scanning. The transforaminal approach and the interlaminar approach through the parasagittal oblique view were performed as described previously [9, 12]. The main points of the near-spinous process view are briefly summarized as follows. Initially, the L5–S1, L4–L5, and L3–L4 intervertebral spaces were identified through the right parasagittal oblique view by starting from the sacrum and moving the transducer cephalad (Fig. 1a). The spinal canal was visible in this view (between the posterior dura and anterior dura). For patients who underwent spine surgery, we selected the intervertebral space with a reserved channel for lumbar puncture. Then, parallel to the spine, the probe was moved toward the median plane to reveal the spinous process (Fig. 1b). Finally, the probe was moved slightly toward the parasagittal view; however, it did not reach the parasagittal plane until the spinal canal was displayed; this view was defined as the near-spinous process view (Fig. 1c). The intersection of the interspinous space and the near-spinous process view was the puncture point (Fig. 1d). After preprocedure scanning, the operator covered the probe with a sterile cover and attached a puncture frame, whose angle was adjusted according to the previously defined puncture path. After the skin was disinfected with iodophor, local anesthesia was introduced using 2% lidocaine. Using iodophor as the couplant for the probe to maintain sterility, the operator scanned again to find the previously defined puncture route and then installed an 18G, 180 mm percutaneous transhepatic cholangiography puncture needle on the puncture frame. The operator’s left hand was used for holding the probe for real-time positioning and the right hand for gradually inserting the puncture needle along the set angle (Fig. 1e). When the US showed that the needle tip reached the spinal canal, the needle core was removed. Cerebrospinal fluid outflow indicated a successful puncture. Thereafter, 5 ml of cerebrospinal fluid was removed, and 5 ml of nusinersen was injected for over 1–2 min. After needle removal, the puncture point was compressed to prevent hemostasis for 20 min. Then, the patient was placed in a comfortable supine position for at least 4 h. If the puncture was unsuccessful, the operator would withdraw the needle and then reinsert it. If it failed thrice, the operator would switch to another intervertebral space. If the puncture failed in three intervertebral spaces or if the patient wished to stop the procedure, the operator would discontinue the procedure and use CT-guided puncture instead.

### Data collection

The number of needle insertions during each puncture was recorded. We defined the operation time as the time from the preprocedure scanning to the completion of the intrathecal injection, and the puncture time as the time from the period the needle punctured the skin to the period of cerebrospinal fluid outflow. Adverse events during lumbar puncture were recorded, and postprocedural adverse events were evaluated through a telephone interview 72 h after puncture. In addition, patients’ motor function before the administration of each dose of nusinersen was assessed using the Children's Hospital of Philadelphia Infant Test of Neuromuscular Disorders (CHOP-INTEND; score range: 0–64).

## Results

**Fig. 2:**
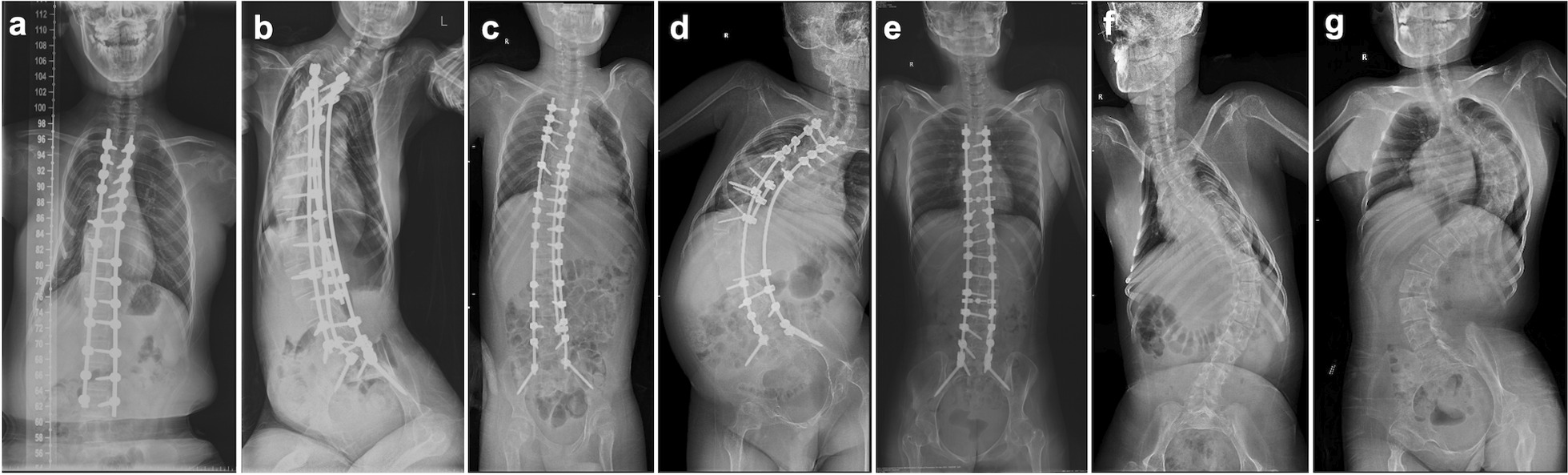
Anteroposterior full-body X-rays of SMA patients with spinal fusion or severe scoliosis. **a**–**g** were for cases 1–7, respectively

Seven patients received intrathecal injections of nusinersen under US guidance. Table 1 summarizes the demographic and clinical data. Two patients were diagnosed with type 1c SMA, 3 with type 2a SMA, and 2 with type 3a SMA. Cases 1–5 had undergone spinal fusion, of which three had additional laminotomy at the L3–L4 level. In the other two patients, although no laminectomy was performed, the L3–L4 or L4–L5 level were purposely exempted in bone grafting. The median time from spine surgery to first nusinersen dose was 5 months (range: 1–23 months). Full-body spine X-rays showed a median Cobb’s angle of 45° (10°–87°), with lumbar spine rotation ranging from grades 0 to IV (Fig. 2).

**Table 1:** Demographics, clinical characteristics, and details of intrathecal injections

Clinical characteristics	Case 1	Case 2	Case 3	Case 4	Case 5	Case 6	Case 7
Age at first dose (y)	12	12	12	19	16	13	15
Sex	Male	Female	Male	Male	Female	Female	Female
Copies of SMN2 gene	3	3	NA	3	3	2	NA
SMA subtype	2b	1c	3a	2b	3a	1c	2a
Motor function	Sit independently	Sit on the back	Sit independently	Sit independently	Sit independently	Sit on the back	Sit on the back
Spine features	Spinal fusion	Spinal fusion	Spinal fusion	Spinal fusion	Spinal fusion	Severe scoliosis	Severe scoliosis
Lumbar laminotomy	No	L3–L4	No	L3–L4	L3–L4	–	–
Time-interval between spine surgery and first dose nusinersen	5 months	1 year and 11 months	1 month	3 years and 4 months	1 month	–	–
Cobb’s angle	13°	45°	10°	87°	18°	77°	75°
Rotation of spine^a^	0	IV	0	IV	I	III	IV
No. of procedures	4	4	4	1	1	4	1
Injection level	L4–L5	L3–L4	L3–L4	L3–L4	L3–L4	L4–L5	L5–S1
No. of interspace level adjustments per procedure	1/1/1/1	1/1/1/1	1/1/1/1	1	1	3/1/1/1	1
No. of insertions per procedure	2/2/1/1	3/2/1/1	2/1/1/1	1	1	8/2/1/1	2
Operation time (min)^b^	35/34/18/19	45/28/17/15	20/18/14/13	17	19	56/37/17/15	37
Puncture time (min)^c^	12/8/5/3	20/10/3/2	7/3/2/2	3	3	30/15/3/3	5
Adverse events	Transient leg numbness, postpuncture headache	Post-puncture headache	No	No	No	Lumbar pain	No
CHOP INTEND score before first and last dose	29/36	19/30	29/37	NA	NA	21/26	NA

*SMA* spinal muscular atrophy, *SMN* survival motor neuron, *NA* not available

^a^Rotation of the spine was referred to as the maximum degree of lumbar spine rotation on X-ray, according to the Nash and Moe criteria

^b^Operation time was defined as the time from the preprocedure scanning to the completion of the intrathecal injection

^c^Puncture time was defined as the time from the needle puncturing the skin to the outflow of cerebrospinal fluid

**Fig. 3:**
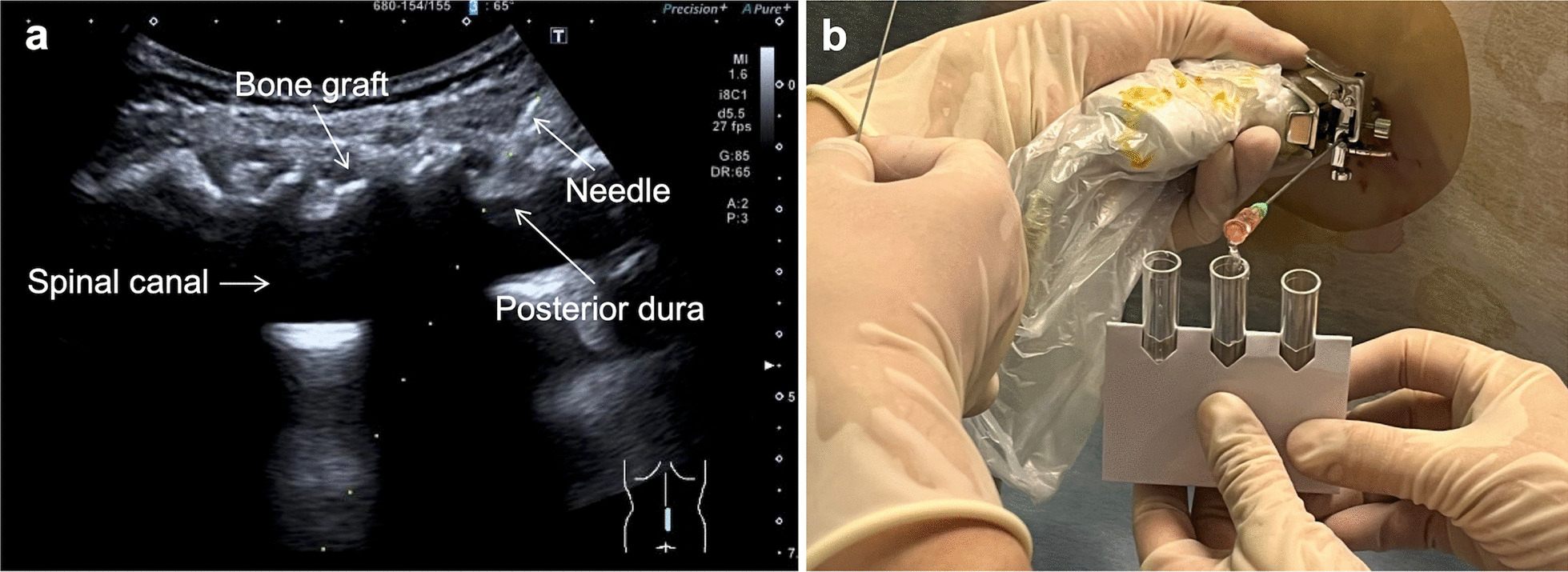
Real-time ultrasound-guided lumbar puncture in an SMA patient who had undergone spine fusion. **a** The ultrasound image showed that the needle reached the spinal canal in the intervertebral space reserved during spinal fusion. **b** Clear cerebrospinal fluid was successfully discharged from the puncture needle

This study performed 20 US-guided lumbar punctures, of which 19 (95%) were successful (Fig. 3). Four patients received four loading doses under US guidance. The other three cases (4, 5, and 7) had previously undergone CT-guided punctures. Case 4 successfully received the fourth dose under US guidance, and case 5 received the fifth dose. For case 7, the third dose was successfully injected under US guidance, but the fourth dose failed. The subsequent CT-guided puncture was successful. All patients tolerated the procedures without requiring sedation or general anesthesia.

For cases 1–5, who had undergone spine surgery, we selected the intervertebral space with a designated puncture channel: L3–L4 for four patients and L4–L5 for the other patient. For case 6, who had severe scoliosis and did not receive surgery, the lumbar punctures at both the L2–L3 and L3–L4 levels were unsuccessful because of severe spinal deformity; finally, we chose L4–L5 with the smallest rotation angle and succeeded. Therefore, the L4–L5 intervertebral space was always selected for case 6 in subsequent procedures, and all were successful. Similarly, the L5–S1 intervertebral space with the smallest rotation angle in the lumbar spines was selected for case 7; the procedure was successful at the third dose but failed at the fourth.

Regarding puncture approach, for case 1, the transforaminal approach was used for the first two procedures, but transient right leg numbness occurred during the second puncture. Therefore, the interlaminar approach was used in the subsequent lumbar punctures of all patients. For cases 2 and 6, the parasagittal oblique view was selected on the first attempt, but it turned out to be difficult; the needle was reinserted several times before the procedure became successful. Hence, we chose the near-spinous process approach (see “Methods” and Additional file 1: Video 1) for the subsequent 16 lumbar punctures, 15 of which went well, except for the fourth dose in case 7. To summarize, of the 19 successful lumbar punctures in this study, two were through the transforaminal approach, two were through the parasagittal oblique approach and the remaining 15 were through the near-spinous process approach.

In 17 (89.5%) out of 19 punctures, the number of insertions was no more than two. More than two insertions (both via the parasagittal oblique view) occurred only in the first dose of cases 2 and 6. The median operation time was 19 min (13–56 min), and the median puncture time was 3 min (2–30 min). With increased experience, the operator performed puncture at a shorter time for the last doses compared with the first dose.

No major adverse events transpired in all procedures, but some minor complications occurred. For instance, 1 patient reported transient leg numbness at the second dose, 2 suffered from a postpuncture headache, and 1 complained of low back pain; nevertheless, all of these were relieved after bed rest within 72 h. Cerebrospinal fluid tests, complete blood count, liver and renal function, and coagulation profile remained unremarkable.

During follow-up, the CHOP-INTEND scores of patients showed varying degrees of improvement after four doses (median, 7.75 [range: 5–11]) (Table 1).

## Discussion

In this study, we introduced a modified interlaminar approach using real-time US guidance to effectively and safely assist intrathecal injection in patients suffering from SMA with spine surgery or severe scoliosis.

Intrathecal injection is difficult for patients with concurrent SMA and severe scoliosis, and it is even more challenging for patients who underwent spinal fusion because of the lack of spinous processes as anatomical landmarks. CT guidance and fluoroscopic guidance are often used for these patients to assist with lumbar puncture [2–4], but long-term radiation exposures are expected [6], especially for children [7]. Currently, reports of US-guided intrathecal injection of nusinersen are still few (Table 2) [9–13], possibly because of technical challenges. Such reports are even fewer in patients with SMA with spinal fusion [11, 12]. Snoj and Salapura [12] reported the use of the transforaminal approach for nusinersen administration in patients with SMA with spinal fusion, but this approach carries a higher risk for radicular and vascular injuries than the traditional interlaminar approach [15, 16]. In recent years, orthopedic surgeons have routinely performed laminotomy during spinal fusion to create a channel for lumbar punctures [17, 18], making it possible to perform intrathecal injections through the interlaminar approach. Veiga-Canuto et al. [11] were the first to report the US-guided interlaminar approach for nusinersen administration for patients who underwent spinal fusion, and our study is the second to report its successful use in five postoperative patients, thereby strengthening the evidence and providing more technical details. Therefore, as long as puncture access is created intraoperatively, US guidance effectively assists the intrathecal injection of nusinersen in patients after spinal fusion.

**Table 2:** Summary of clinical features of the present and published studies associated with ultrasound-guided nusinersen administration

References	Nagano et al. [13]	Zhang et al. [9]	Zanfini et al. [10]	Veiga-Canuto et al. [11]	Snoj et al. [12]	This study
No. of patients	1	3	18	18	14	7
Age, years	21	28 (14–34)	49 (21–66)	16 (7–63)	33 (22–50)	13 (12–19)
Children: adults	0: 1	1: 2	0: 18	10: 8	0: 14	6: 1
*SMA subtypes*						
Type 1	0	0	0	0	0	2
Type 2	1	1	7	14	11	3
Type 3	0	2	11	4	3	2
*Spine surgery*						
Growing rods (No.)	Previously implanted and removed later	–	–	9	–	–
Spinal fusion (No.)	–	–	–	3	10	5
Cobb’s angle: median (range)	60°	(103°–130°)	(30°–> 50°)	53.5° (21°–87°)	54.6° (20°–85°)	45° (10°–87°)
Lumbar spine axial rotation	Severe	NA	Mild-severe	NA	43.2 (10–81)	Mild-severe
No. of successive procedures	3	15	57	91	14	19
Success rate	100%	100%	100%	96.8% in procedures 77.7% in patients	60% in patients	95%
Injection levels	L3–L4, L4–L5	L4–L5, L5–S1	L3–L4, L4–L5	NA	L1–L2, L2–L3, L3–L4	L3–L4, L4–L5, L5–S1
Approach	Parasagittal oblique approach	Parasagittal oblique approach	Parasagittal oblique approach	Parasagittal oblique approach	Transforaminal approach	Near-spinous process approach
Sedation	Sedation with midazolam	No	No	Sedation with midazolam and Ketamine	No	No
*Adverse events*						
Post-puncture headache (°)	No	1	2	3	1	2
Severe adverse events	No	No	No	Acute urinary retention	Inadvertent vein access	No

*SMA* spinal muscular atrophy

In selecting the intervertebral space for lumbar punctures, we chose the interlaminar space with a designated puncture path for postoperative patients, and the interspace with the smallest rotation angle of adjacent vertebral bodies for patients without surgery. In case 6, the lumbar puncture failed at the L2–L3 and L3–L4 levels at first, but it succeeded at the L4–L5 level with a smaller rotation angle. The reason is that the greater the spine rotation angle, the more inclined the puncture needle would be, and the more difficult it is to control the direction of the needle. To ensure accuracy of the needle direction, we used a puncture frame to fix the needle direction during the lumbar puncture, which also made it easier for the sonographer to operate the probe and the puncture needle simultaneously [8]. However, US guidance remains challenging for patients with a very narrow intervertebral space because of severe rotation and scoliosis. For case 7, the third nusinersen dose under US guidance was successful, but the fourth dose failed. Therefore, for patients with severe spinal deformities, US guidance can be tried first. If it fails, CT guidance is an alternative option. In addition, although nusinersen was injected at different levels, no differences in efficacy and safety were found in this study.

According to the anatomical characteristics of severe spinal deformities and our experiences, we proposed that the near-spinous process view is a better US-guided interlaminar approach for lumbar puncture than the parasagittal oblique view. Traditionally, lumbar puncture is performed from the spinous process plane, where the intervertebral space is widest. However, acoustic shadows appear under the spinous processes on US; consequently, the spinal canal cannot be clearly visualized. Therefore, the parasagittal oblique view, which is approximately 1–2 cm away from the spinous process view, is mostly used during US guidance to better visualize the spinal canal [19, 20]. However, the intervertebral space of the paramedian approach is relatively narrow; this limited width can be aggravated by severe scoliosis and/or rotation. In our study, the first dose via the parasagittal approach for patients 2 and 6 was unsuccessfully administered because of the narrow intervertebral space. Thus, we designed a near-spinous process view obtained by moving the probe slightly outward from the spinous process until the spinal canal could be visualized. This view has not yet reached the standard laminar view, so the intervertebral space is wider than the parasagittal oblique view, making lumbar puncture easier to perform. In this study, 15 subsequent lumbar punctures were successfully performed through the near-spinous process view. Following the standard protocols, even our young sonographers could perform US-guided lumbar punctures independently. Therefore, according to our single-center experiences, the near-spinous process view may be a good choice for US-guided intrathecal injection in patients with spinal deformities. However, this approach still needs to be confirmed by the experience of more centers and larger samples.

In the present study’s preprocedure preparation, full-body spine X-rays were sufficient for assessing the degrees of scoliosis and spine rotation, and 3D-CT reconstructions, as reported in the previous literature [9, 13], were not required because sonographers relied primarily on real-time ultrasound imaging rather than CT for lumbar puncture. Previously, the puncture path designed according to CT was unsuccessful in the actual US-guided puncture process [9]. Preventing preprocedure CT also reduces patient’s exposure to radiation. In addition, pediatric patients who underwent US-guided intrathecal injections were mostly sedated [11]. In our study, all patients, who were above 10 years old, received intrathecal injection under local anesthesia and cooperated well, suggesting that older children can tolerate US-guided puncture without the need for general anesthesia or sedation.

This study has some limitations. One is that the patients in this study were all teenagers. The feasibility of the near-spinous process view in adult patients still needs to be validated in the future. Another limitation is the use of 18G puncture needles instead of 20G or 22G needles reported in previous studies [9, 11, 12]. In this study, no serious adverse events occurred, and minor complications were similar to those previously reported lumbar puncture complications, such as postpuncture headache.

## Conclusions

Real-time US guidance is safe and effective; hence, it may be the preferred imaging-assisted technique for intrathecal injections in patients with SMA with spine surgery or severe scoliosis. This study provides detailed steps of the US-guided intrathecal injection of nusinersen and proposes that the near-spinous process view can be used as a modified interlaminar approach.

## Supplementary Information

**Additional file 1**. Video S1: The procedure of ultrasound-guided intrathecal injection of nusinersen.

## Data Availability

The data that support the findings of this study are available on request from the corresponding author.

## Abbreviations

SMA: Spinal muscular atrophy
CT: Computed tomography
CHOP-INTEND: Children's Hospital of Philadelphia Infant Test of Neuromuscular Disorders
US: Ultrasound

## References

[1] Mercuri E, Darras BT, Chiriboga CA, Day JW, Campbell C, Connolly AM (2018). Nusinersen versus sham control in later-onset spinal muscular atrophy. N Engl J Med.

[2] Spiliopoulos S, Reppas L, Zompola C, Palaiodimou L, Papadopoulou M, Filippiadis D (2020). Computed-tomography-guided transforaminal intrathecal nusinersen injection in adults with spinal muscular atrophy type 2 and severe spinal deformity. Feasibility, safety and radiation exposure considerations. Eur J Neurol.

[3] Cox M, Atsina KB, Ramchand P, Ji J, Sedora-Roman N, Pukenas B (2021). Computed tomography-guided transforaminal lumbar puncture using local anesthesia and a straight 22-gauge spinal needle for intrathecal nusinersen in adults: findings in 77 procedures. Interv Neuroradiol.

[4] Jacobson JP, Cristiano BC, Hoss DR (2020). Simple fluoroscopy-guided transforaminal lumbar puncture: safety and effectiveness of a coaxial curved-needle technique in patients with spinal muscular atrophy and complex spines. AJNR Am J Neuroradiol.

[5] Salapura V, Snoj Z, Leonardis L, Koritnik B, Kostadinova V (2022). Cone-beam computed tomography guided nusinersen administrations in adult spinal muscular atrophy patients with challenging access: a single-center experience. Radiol Oncol.

[6] Kizina K, Stolte B, Totzeck A, Bolz S, Fleischer M, Monninghoff C (2019). Clinical implication of dosimetry of computed tomography- and fluoroscopy-guided intrathecal therapy with nusinersen in adult patients with spinal muscular atrophy. Front Neurol.

[7] Pearce MS, Salotti JA, Little MP, McHugh K, Lee C, Kim KP (2012). Radiation exposure from CT scans in childhood and subsequent risk of leukaemia and brain tumours: a retrospective cohort study. Lancet.

[8] He XB, Liu YY, Huang GM, Du D (2020). The clinical application of puncture frame in establishing ultrasound guided percutaneous nephrolithotomy access. Urol J.

[9] Zhang J, Cui X, Chen S, Dai Y, Huang Y, Zhang S (2021). Ultrasound-guided nusinersen administration for spinal muscular atrophy patients with severe scoliosis: an observational study. Orphanet J Rare Dis.

[10] Zanfini BA, Catarci S, Patanella AK, Pane M, Frassanito L, Filipponi E (2021). Ultrasound assisted lumbar intrathecal administration of nusinersen in adult patients with spinal muscular atrophy: a case series. Muscle Nerve.

[11] Veiga-Canuto D, Cifrian-Perez M, Pitarch-Castellano I, Vazquez-Costa JF, Aparici F (2021). Ultrasound-guided lumbar puncture for nusinersen administration in spinal muscular atrophy patients. Eur J Neurol.

[12] Snoj Z, Salapura V (2022). Ultrasound-guided transforaminal approach for nusinersen delivery in adult spinal muscle atrophy patients with challenging access. Muscle Nerve.

[13] Nagano T, Sakura S, Imamachi N, Saito Y (2020). Ultrasound-assisted intrathecal injection of nusinersen in a patient with severe vertebral deformity: a case report. JA Clin Rep.

[14] Nash CL, Moe JH (1969). A study of vertebral rotation. J Bone Joint Surg Am.

[15] Weaver JJ, Hallam DK, Chick JFB, Vaidya S, Shin DS, Natarajan N (2021). Transforaminal intrathecal delivery of nusinersen for older children and adults with spinal muscular atrophy and complex spinal anatomy: an analysis of 200 consecutive injections. J Neurointerv Surg.

[16] Bortolani S, Stura G, Ventilii G, Vercelli L, Rolle E, Ricci F (2019). Intrathecal administration of nusinersen in adult and adolescent patients with spinal muscular atrophy and scoliosis: transforaminal versus conventional approach. Neuromuscul Disord.

[17] Labianca L, Weinstein SL (2019). Scoliosis and spinal muscular atrophy in the new world of medical therapy: providing lumbar access for intrathecal treatment in patients previously treated or undergoing spinal instrumentation and fusion. J Pediatr Orthop B.

[18] Machida S, Miyagi M, Saito W, Matsui A, Imura T, Inoue G (2021). Posterior spinal correction and fusion surgery in patients with spinal muscular atrophy-associated scoliosis for whom treatment with nusinersen was planned. Spine Surg Relat Res.

[19] Yoo S, Kim Y, Park SK, Ji SH, Kim JT (2020). Ultrasonography for lumbar neuraxial block. Anesth Pain Med (Seoul).

[20] Grau T, Leipold RW, Horter J, Conradi R, Martin EO, Motsch J (2001). Paramedian access to the epidural space: the optimum window for ultrasound imaging. J Clin Anesth.

